# Melancholia

**Published:** 1898-09

**Authors:** Lionel A. Weatherly


Medico-Chirurgical Journal.

SEPTEMBER, 1898.

### MELANCHOLIA.

BY

Lionel A. Weatherly, M.D., C.M. Aberd., M.R.C.S. Eng.

Melancholia is a disease unique in the fact that every human being who has reached that stage of his existence which can be called the battle of life can enter into and appreciate to a greater or less degree the subjective sensations of the sufferer from this complaint, by reason of having, however temporarily, passed through periods of mental depression himself. Lucky indeed is the man who can assert in all sincerity that he has never known what it was to be depressed. To those of the most hopeful and sanguine temperament there must come times of worry, sorrow, anxiety, and trouble which cannot be quickly or lightly thrust on one side. In the still and silent watches of the night, when all the vital processes are at their lowest ebb, with sleep banished, and the worrying mind intense in its activity, the weary hours all the more irksome and hard to bear from the impossibility of being "up and doing"—then it is that one can have some faint idea of the tortures the unhappy sufferer from the disease under discussion has to endure.

But depression is not necessarily melancholia, though it is its most prominent symptom. Dr. Mercier defines melancholia as a disorder characterised by a feeling of misery which is in excess of what is justified by the circumstances in which the individual is placed.[1] This definition is distinctly good as applied to simple melancholia without delusion, but does not hold good when delusion is present; for all must recognise that in many cases of melancholia with delusion, if the false ideas were true, the depression would be little more than we might reasonably expect. It is therefore well to add to that definition, "*or* a feeling of misery resulting from delusion."

The pathology of melancholia may be generally said to be defective nutrition caused by faulty blood-supply to the brain, either in quantity or quality, which produces exhaustion or poisoning of the brain-cells. I am now dealing with melancholia as a disease *per se*, and not as a transitory symptom of other forms of insanity.

We may have this disease acute, subacute, or more or less chronic. We may have it without delusion or with definite false ideas, and these may be of every sort and kind. We may have with it very distinct active suicidal symptoms, or we may only be suspicious of the suicidal tendency from the nature and gravity of the depression. In the large majority of cases of melancholia, unless successfully treated in the early stage, delusions sooner or later show themselves; and the evolution of delusion in many of those cases is, to my mind, most interesting and has a very important bearing on the ever-urgent necessity of early treatment.

Though we may all agree that to build up the health of the patient, to rectify, if possible, any physical ailment, either medical or surgical, to procure sleep, and to alter the gloomy feelings by change of thought and by occupation of mind and body must be the leading lines upon which our treatment must be based, the mode of applying this cannot be so definitely stated. Each case must be treated after the most careful investigation into its history, the causes, if traceable, the habits and feelings of the patient, and the means at disposal. The last plays often a most important part in the mode of treatment to be adopted.

With regard to physical disease as an exciting cause of

[1] *A Dictionary of Psychological Medicine*, edited by D. Hack Tuke, 1892, vol. ii, p. 787.

melancholia, phthisis stands first on the list, and I need not waste space by suggesting the necessary medical treatment for this class of case. Disease of any of the internal organs of the abdomen may, and often does, produce actual melancholia, without any other assignable cause, excepting possibly the predisposing one of hereditary taint. Thus stone in the kidney, floating kidney, impacted gallstones, diseases of liver, pancreas, stomach, spleen, uterus, and ovaries, hemorrhoids, and papillomatous growths in the rectum have all been observed by myself as exciting causes of this mental disease. The immediate necessity of diagnosing those causes and doing our best to remove them is, of course, too patent to need more than this passing reference to them.

Sleeplessness is, as a rule, one of the very earliest symptoms of melancholia, and it is of the very highest importance that it should be relieved as early as possible. In a large number of cases, the patient will say he cannot sleep because the brain seems so active with all his worries and anxious thoughts. The arterial supply to the brain is not sufficiently inhibited to bring it down to the level which is obtained in normal sleep. Before trying drugs for this, I strongly advise other means. Exercise in the open air of all kinds, such as walking, riding, bicycling, tennis, should be enforced at stated times during the day, making each such exercise to fall definitely short of fatigue-point. This fatigue-point, whether mental or physical, is a most important consideration to notice in the treatment, and too much stress cannot be laid upon keeping well behind this point in all the occupations suggested. Then some easily-digested food before retiring to rest will often greatly relieve (1) by diverting the blood from the cerebral area; (2) *after* digestion has taken place, by nourishing the brain-cells. A hot bath before going to bed is also often very efficacious, as well as local stimulation of the cutaneous surface by mild mustard plasters the warm pack, or abdominal compresses. A tumbler of hot water or hot milk and water, taken after getting into bed, will also in the same way be of service in the early treatment of sleeplessness. The drug, I believe, best suited and least harmful for sleeplessness from an over-active wearied brain, is phenacetin in 10 or 15 grain doses, repeated in an hour's time if necessary. It seems to take, so to say, the rough edge off their mental activity; so that a quiet dozy feeling soon develops into absolute sleep, and with no bad after-effect. Of course if all mild measures fail, recourse must be had to soporifics. Opium alone I do not like; neither do I care for chloral by itself. The mixture of the two often suits well, but should, I maintain, be varied with other drugs. Sulphonal must be cautiously given, and the effects carefully watched; and I am convinced that this is a medicine which should not be long continued, more especially if the dose has to be increased to procure sleep. Paraldehyde is in some cases most useful; in others, a failure. Trional the same. Dr. Clouston advocates a mixture of Tincture of Cannabis Indica ♏ x., and Potassium Bromide gr. xx.; but as he so justly says, "We have not yet discovered a perfect narcotic that gives brain-quiet combined with increased appetite and body weight."[1]

Once recognise that a patient is in danger of melancholia, I think we may truthfully say that in the large majority of cases change in the surroundings is of the highest importance. What this change should be must be determined in each individual case; and if possible the patient's own feelings should be consulted. To one, rest from work, a quiet social life with simple amusements and occupation will do wonders; while to another, an active life of sight-seeing, within limits, will quickly prove beneficial. A sea-voyage is a plan not to be advised without careful thought, and as a rule it is contra-indicated in melancholia; for, as Dr. Blandford has pointed out, it is a monotonous existence, there is little to distract the thought, and there is an ever-present danger of suicide. In cases in which the patient's mind is engrossed by worries and troubles, fanciful or otherwise, intimately connected with his work and his family, it is essential that he be sent from home as quickly as possible. No doubt the serious question, in all these cases in which change is imperative, is where to send the patient. His relatives do not consider him insane enough to send him to an asylum, and yet they cannot, perhaps, afford sufficient for

[1] *Clinical Lectures on Mental Diseases*, 3rd Ed., 1892, p. 128.

him to be properly looked after in a private family. Proper private treatment means of necessity expense; and as long as the law does not allow anyone to receive more than one person of unsound mind under their charge, so long must such a mode of treating the early stages of insanity be only applicable to those with means at their command. Let us hope it will not be long before we have some system of "half-way houses" in which cases can be received, properly looked after, and treated on terms within reasonable reach of all.

It is not, however, merely for the benefit of change from the usual environment of the patient that we must, as a rule, insist upon his removal from home surroundings. It is, to a very large extent, because in his own home it is almost impossible to carry out that regular system of exercise, occupation, and feeding which alone will lead to the hoped-for rapid recovery. On this question I shall have more to say later on, but I may here at once state my firm conviction that once melancholia has definitely developed, unless the means allow of his being placed in a private family in which the right systematic treatment of his case with the adequate supervision always so necessary can be carried out, it is by far the best for the relatives, for the medical attendant's peace of mind, and for the patient himself that he be sent to an asylum. There can be no doubt that the average length of time melancholics remain in asylums before being thought fit for discharge, would be very considerably lessened if they were sent to those institutions much earlier than is the general rule.

At the 1895 meeting of the British Medical Association a most interesting discussion took place on the question of "Rest *v.* Exercise" in the treatment of melancholia. Dr. (now Sir) John Batty Tuke, on the one side, advocated most earnestly rest in bed, massage, and constant feeding for a large proportion of sufferers from this disease, and certainly supported his contention with some rather startling statistics; whilst Dr. Clouston, on the other side, deprecated this form of treatment, except in rare cases of great physical weakness with great trophic disorder, and argued, to my mind upon the most rational grounds, that open air and exercise must always be the most potent factor in the successful treatment of this complaint.

Agreed that we want in all these cases to feed up, to nourish our patient's body, do we not aim at doing more than this? Do we not desire to lessen the chances of constant introspection and to increase and encourage observation of things entirely outside their miserable ideas and gloomy forebodings? It is only by occupation of mind and body that one can hope to do this, and thus give the mind that true rest which it requires. The word "rest" is not properly appreciated by the ordinary individual. To the melancholic great activity of the mind, if that activity is directed away from his depressed ideas and feelings, is after all the greatest possible rest. For my own part, I feel greatly handicapped in my treatment of a case of melancholia if I have to realise that the patient's physical condition renders rest in bed a necessity.

With regard to exercise and occupation, a great deal depends upon our patient's habits, hobbies (if any), and physical strength; but, generally speaking, the plan I have always found most beneficial is to map out the patient's day for him, with a more or less varied programme of short periods of exercise in the open air of some kind, alternating with short periods of reading; games of all sorts, such as billiards, cards, draughts, chess; or other occupations, such as drawing, painting, setting music, according to the patient's inclinations. Every effort must be made to make him concentrate his thought as much as possible on whatever he is at the time doing, and directly the exercise or occupation is apparently getting irksome to him to leave it off and start something else.

Of all exercises in the open air, I look upon cycling as by far the best for those melancholics who are not definitely suicidal. It is a means of exercise, of getting plenty of fresh air, and necessitates such an amount of constant attention that introspection must be at its minimum. Added to this, we have the pleasurable sensation of going through the air at a fairly good pace, and the interest of seeing fresh scenery, etc.

Golf is, again, a capital form of exercise; but I do not greatly advocate it unless the golf-links are very near the patient's residence, as otherwise it does not admit of those short periods of exercise in the open air, varied with other occupations, which I so strongly advise. During convalescence, I can well imagine that it is a most useful and beneficial way of spending a few hours.

The man who does not care about games, who hates music, who takes no interest in reading, has no hobbies outside his regular work, and who becomes a melancholic, is a very difficult patient to treat.

The one ever-present symptom in melancholia is a disinclination for food. It may be a mere want of desire to take nourishment, or it may be an absolute distaste for it, or a positive refusal to take it either from some delusion as to its being wrong and wicked or that there is poison in it; or, again, with a definite determination to commit suicide by starvation. It is of the most vital importance that we should recognise that our great aim in melancholia should be directed to stimulating and increasing the activity of the processes of nutrition. Don't let us for one moment allow ourselves in these cases to lessen the quantity and quality of food in consequence of what we may think is a dyspepsia. We may easily be making a fatal mistake. Food, and an abundance of it, must be administered in these cases, looking, of course, to the important points of choosing the most digestible forms of nourishment and helping forward by all means in our power its proper assimilation. We should look to the proper mastication of food, to mouth disinfection, to the correction of any stomachic defect, and should guard against an excess of meat dietary, with its danger of toxic re-absorption from undigested and decomposing particles. The dietary should be rich in fats. Let milk be our sheet-anchor, and add to it fish, fowl, ham, bacon, eggs, and farinaceous diet.

I cannot too strongly impress upon all medical men the necessity, in these cases of melancholia, of having the most strict account kept of the amount of nourishment the patient is taking, and not simply trusting to the bare statement of the relatives: "He has done fairly well to-day." If possible, he should be weighed pretty frequently; and if loss of flesh, however slight, is still persisting, some other measures of feeding should at once be adopted. The artificial feeding of patients may be of great value in saving the life of an emaciated melancholic who has been allowed to drift into this condition, but why should it not be a mode of much earlier treatment? I have no hesitation in saying that the great value of this form of feeding consists in its early application. That it is a difficult matter for a general practitioner himself to feed a patient regularly and frequently enough, is only too true. All the more reason then for him to refuse to treat such a case any longer, and insist upon its being sent to an institution in which such feeding can be properly carried out. Space does not admit of my going as exhaustively as I should like into this question of feeding in melancholia. That it is of the utmost importance I maintain, and its early recognition will save many a melancholic's life and shorten the duration of his mental illness to a very considerable extent.

In about 65 per cent. of melancholic cases suicidal tendency is manifested, but it may be wiser to always fear the possibility of suicide in all cases of mental depression. If a definite suicidal impulse is present, then no time should be lost in placing the patient under the most stringent supervision, and an institution is the only place where this can be properly done, unless, of course, expenditure is a matter of indifference. Even in an asylum, with every conceivable safeguard, with a staff of watchful attendants and nurses, it is impossible to prevent melancholics from committing suicide, and they do it in such a way that the most careful investigation can throw no blame upon anyone. How much more difficult must it be to guard against such a contingency in a private house?

We must do all in our power to gain the confidence of the patient. We must never deceive him or allow those in charge of him to do so. It is absolutely wrong policy for an attendant or nurse in these cases to get their patients to do what they wish by false promises or by agreement with their insane ideas and arguments. The quicker we can get a patient to recognise that he must be obedient, as far as he is able, to our orders and requests, the better it will be; and there can be no doubt that the value of asylum treatment for many of these cases consists in the atmosphere of order and discipline in which the patient finds himself. The individual discipline may have been irksome to him, may have been actually productive of a determinate obstinacy against carrying out the wishes of those who attempt to exercise it; whereas the general discipline of an institution does not seem in the least to irritate him, and he falls in with it as a matter of course.

Many melancholics, too, are intensely selfish. By the nature of their unhappy complaint they can think of nobody but themselves, and will be constantly trying to attract attention and to gain the sympathy of those about them by a never-ceasing recital of all their woes. It is different with such a case in an institution; and Dr. Clouston once asked a melancholic who had been very ill and unmanageable at home, and who had improved greatly after a few days in the asylum, what had made him better. His reply was: "I found myself among a lot of people who did not care a farthing whether I was miserable or not, which made me angry, and I got well."[1]

But should we content ourselves, when we have got our patient well, with patting him on the back and congratulating him on his recovery? Surely not; for do we not realise the truth of the assertion that melancholia far more often results from innate brain constitution than from outside calamities and afflictions? Thus, a man who has once been a sufferer from this disease is always likely to drift into it again, unless all means be taken to prevent such a recurrence. It may be that the work which our patient has been doing is unsuitable to his mental constitution. If so, at all hazards he must alter it. If he has had no hobbies, let him at once initiate one or more. Let him, above all, see that he keeps a sufficiency of fat on him. Let the falling off of even a few pounds in weight be to him a danger signal. Let him do nothing in big spurts, and let him strenuously avoid, whether in pastime or in work, that point, so different in each of us, and even so different in the same person at different times, namely, fatigue-point.

Much have I left unsaid. It is a subject upon which a volume might be written. The difficulties which beset those who have to treat the sufferers from this disease are great. I

[1] *Op. cit.*, p. 126.

shall be satisfied if I have said anything which will help forward the recovery of our unfortunate brothers and sisters whose mental constitution has rendered them prone to suffer from this most sad affliction.

